# ‘It is a lifeline’: International cross-sectional survey of benefits, barriers and acceptability of online yoga during the COVID-19 pandemic

**DOI:** 10.1371/journal.pone.0341852

**Published:** 2026-02-18

**Authors:** Tina Cartwright, Lindsay Metcalf, Vipin Wadhen

**Affiliations:** 1 Psychology, School of Social Sciences, University of Westminster, London, United Kingdom; 2 Psychology, School of Law, Social and Behavioural Sciences, Kingston University London, United Kingdom; Marwadi University, INDIA

## Abstract

**Objectives:**

Yoga is associated with physical and mental health benefits. Online delivery may increase access to these benefits, yet limited research has examined its effectiveness and acceptability. This cross-sectional study explored the perceived benefits, barriers, and acceptability of online yoga among a large, global sample of practitioners and teachers during the early phase of the COVID-19 pandemic. A secondary aim was to assess the psychosocial health of practitioners.

**Methods:**

A total of 1,185 participants (511 yoga students, 586 yoga teachers) completed an online survey assessing sociodemographic characteristics, yoga dosage, perceived benefits and barriers, and psychosocial health indicators (social isolation, depression, anxiety, and stress). Open-ended responses were analyzed using inductive thematic analysis to identify perceived challenges and benefits of online yoga.

**Results:**

Participants reported low levels of stress, anxiety, and depressive symptoms compared to general population norms during the pandemic. Hierarchical regressions showed small but significant associations between yoga practice and mental health. Controlling for age and gender, days per week was associated with lower depression (β = −.10, p = .011) and years of practice with lower anxiety (β = −.07, p = .045). Age was inversely associated with depression, anxiety, and stress. Both quantitative and qualitative data indicated that the benefits of online yoga outweighed its barriers. Key reported benefits included social connectedness, increased accessibility and convenience, improved mental and physical health, and opportunities for personal practice. Reported barriers included reduced group connection, limited personalized guidance, and practical challenges related to technology and the home environment.

**Conclusions:**

This mixed-methods study offers novel insights into the perceived effectiveness and limitations of online yoga from both student and teacher perspectives. Interpreted through the COM-B model, findings highlight key barriers and facilitators that can inform the future design and delivery of online and hybrid yoga interventions in a post-pandemic context.

## Introduction

Yoga is a multicomponent holistic practice increasingly popular in the Western world to promote physical and mental wellbeing. It is estimated that around 31 million US adults have tried yoga [1], and it has continued to grow in popularity worldwide [2]. Research in the United States, Australia and Europe consistently find that yoga practitioners are more likely to be female, white and well-educated [1,3–5], with recent research exploring ways in which to increase access beyond this narrow demographic [6–8]. Compared with physical activity, yoga incorporates a range of components that go beyond physical fitness goals, including postures, breathing techniques, mindfulness and relaxation. For this reason, yoga has been described as a ‘holistic movement practice’ (HMP) along with other mind-body practices, that purposively support mental health and wellbeing [9].

Indeed, several systematic reviews support the effectiveness of yoga interventions for the management of stress [10,11], anxiety [12], depressive symptoms [13,14] and psychological wellbeing [15]. Yoga is also associated with positive physical and lifestyle outcomes, such as fitness, respiratory functioning, sleep, energy and self-care [16,17]. Various pathways have been proposed to explain the impact of yoga on mental health and wellbeing. Physiological and psychological mechanisms may underpin improved self-regulation and management of stressors, with evidence for changes in brain structure and function [18], down-regulation of the autonomic nervous system [19–21] and increases in neurotransmitters, such as GABA, which are implicated in mental disorders [22,23]. Psychosocial mechanisms such as increased emotional and mind-body awareness, mindfulness and self-compassion enable practitioners to build resilience and adaptive coping responses [16,24], whilst class participation can enhance support networks and social connectedness [25].

The onset of the COVID-19 pandemic in early 2020 had a significant impact on the physical and mental health of the global population, causing considerable disruption to daily lives, including physical distancing, quarantine, and periods of lockdown [26]. Reflecting the unprecedented stress during this period, global surveys revealed a deterioration in mental health compared with pre-pandemic levels, with a spike in mental health problems during the first wave in April-May 2020 [27,28]. The impact on psychological distress, anxiety and social isolation is well documented [27–29]. Given the limited access to face-to-face psychosocial and mental health support, digital and online tools became an increasingly important source of support. In a rapid review of home-based activities to promote wellbeing during the pandemic, yoga was included as a potential tool to improve anxiety, depression and psychological distress [30]. In addition to mental health benefits, yoga has been recommended for those at risk of social isolation given the association with social connectedness, with recent integration into healthcare through social prescribing [25]. During the pandemic, yoga delivered online became the primary means to access yoga globally during periods of lockdown. It is unclear whether the psychosocial benefits of in-person yoga can be realised when delivered online.

Prior to the pandemic, research on the effectiveness and acceptability of online yoga was limited despite the growing popularity of telemedicine [31]. Nevertheless, existing studies have demonstrated a degree of effectiveness and acceptability, warranting further exploration of online yoga as an intervention tool. A systematic review of eight yoga intervention studies found that online delivery was generally feasible and acceptable to participants, providing preliminary evidence for the alleviation of symptoms across a range of health conditions [32]. Online yoga may utilise a variety of delivery methods, with early studies using video recordings demonstrating positive results for health [33,34]. More typically, interventions use recorded classes, using pre-existing [35] or specifically designed interventions [36], or are live-streamed [37]. There is currently no consensus regarding which approaches are most effective or acceptable [32]. Recorded classes offer greater flexibility in terms of scheduling and convenience but lack the accountability and two-way interaction enabled by livestream classes [38]. Whilst offering a cost-effective method to widen access to a larger population, a study of adults with mood disorders showed low adherence and retention when using a recorded yoga class [39]. Conversely, participants taking part in live-stream formats report appreciating the group format and instructor feedback but highlight challenges around scheduling and technological difficulties [38,40].

Several yoga intervention studies conducted during the pandemic demonstrated acceptability and effectiveness of online delivery across a range of populations, including office workers [41], older people [42–44] and patients with ankylosing spondylitis [45,46]. These interventions have also demonstrated high adherence rates, with qualitative feedback suggesting the acceptability of online yoga, even in the face of technological challenges [42,47]. When compared with in-person yoga, studies have broadly found equivalent effects and acceptability [47], although the skill of the yoga instructor appears paramount to creating a sense of safety and community [42]. Few studies have explored perceptions of online yoga in the wider population. A small cross-sectional survey in Australia during the pandemic suggested an overall preference for in-person delivery, despite the greater convenience and affordability of online delivery [14]. The benefits of online yoga for widening access and reducing costs were also highlighted by yoga instructors in an Indian convenience sample, along with concerns about technical difficulties, safety (risk of injury) and loss of social connection [48].

It has been suggested that the increased accessibility afforded by online yoga classes from home may override key barriers associated with in-person yoga classes. Barriers and facilitators to initiating and maintaining both in-person and online yoga practice, can be understood through the integrated model of Capability, Opportunity, Motivation and Behaviour (COM-B) [49]. Theoretical models help to explain and predict health behaviours and are important in designing and evaluating interventions [50]. *Capability* can be psychological or physical. Physical capability can impact on uptake and adherence; for example, adherence to a yoga program was lower among adults with obesity [51]. However, recognising these barriers enables appropriate adaptations, such as offering chair yoga for those with restricted mobility [52]. *Opportunity* can be physical or social, and relates to having the time, space and social support to practice yoga. Physical opportunity is most commonly hindered by family and occupational commitments [53], whilst attending in-person classes requires the time and resources to travel and pay for classes. The increased accessibility and affordability of online classes is thus a key facilitator of opportunity. *Motivation* can be reflective, such as recognizing the value of yoga, or autonomous, driven by intrinsic enjoyment and satisfaction. Practitioners’ commonly report reflective motivations related to mental and physical benefits [1,4,54], and autonomous motivation is positively associated with health behaviour [55].

An additional question regarding online yoga is whether it has the potential to increase yoga practice through encouraging a regular home practice. This is viewed as an essential component of many yoga health interventions [25,56], including effectiveness in reducing depressive symptoms [57]. In a large US survey, having an established home yoga practice predicted better health than yoga dosage alone [58] and frequency of yoga practice consistently demonstrates small but significant relationships with health indices [4].

The pandemic caused a shift towards online yoga usage and created a unique opportunity to survey the yoga community about their experiences. Whilst research has recently begun to evaluate the effectiveness and acceptability of online yoga, most studies have focused on online yoga interventions in clinical or at-risk populations. Few studies have explored yoga practitioners and teachers’ experiences and beliefs about online yoga in the wider global population. This also has important ramifications for understanding the potential benefits and challenges of online yoga, in order to inform future interventions through the lens of the COM-B model. The aims of the current study were therefore to: 1) assess the mental health and social connectedness of yoga practitioners during the first wave of the pandemic; 2) evaluate the relationship between yoga dosage and psychosocial health; 3) investigate the benefits, barriers and overall acceptability of online yoga classes from the dual perspective of teachers and practitioners.

## Methods

### Design and recruitment

The study was a cross-sectional online survey, incorporating measures of psychosocial health, yoga practice and open-ended questions around experiences of online yoga. The survey was hosted on Qualtrics survey software with data collection taking place over a two-month period between 6 May and 6 July, 2020. Participants from any country were eligible to participate if they were at least 18 years of age and had practised yoga in the past 12 months. The survey link was primarily distributed through social media. Yoga studios and organizations in the UK and US (including YogaAlliance, YogaMatters, British Wheel of Yoga, Minded Institute) were contacted and asked to share the link amongst their communities. Following information about the study, participants gave informed written consent electronically and passed inclusion criteria before beginning the survey. The study was approved by the University of Westminster Psychology Ethics Committee (ETH1920−0762 on 04/05/2020).

### Measures

The survey measured sociodemographic variables (age, gender, ethnicity, education, country of residence) and lockdown-specific information, such as living situation (i.e., alone, with parents, with roommates), location (urban, suburban, rural) and employment status during lockdown.

*General health* was measured by a single 5-point Likert-scale measure of self-rated health, where 5 indicates excellent and 1 indicates poor health [59]. For the following scales, Cronbach’s Alpha (α) is reported for this study’s sample.

*Social isolation* was measured by the Hawthorne Friendship Scale (HFS) [60] which includes six 5-point Likert-scale items which measure subjective feelings of isolation and connectedness. Summed scores range from 0–24, where higher scores indicate social connectedness and lower scores indicate isolation (α = .67).

*Negative affect* was assessed using the Depression, Anxiety and Stress Scale (DASS-21; Lovibond & Lovibond, 1995) which consists of 21 four-point (0–3) Likert-scale items divided into three sub-scales designed to measure the emotional states of depression (α = .88), anxiety (α = .78) and stress (α = .86), Summed scores are multiplied by two for the final score of each subscale, where higher scores indicate greater symptom severity.

*Perceived benefits and barriers to yoga* was assessed using a modified version of the Perceived Benefits and Barriers to Physical Activity Scale [61] where “physical activity” was replaced with “yoga”. It consists of ten 5-point Likert-scale items divided into subscales for the barriers and benefits of taking yoga classes in general. The reliability was good for the barrier subscale (α = .71) and moderate for the benefit subscale (α = .65). Seven additional Likert-scale items measured agreement with statements about benefits and barriers of online yoga specifically, developed from previous literature. For example, “online yoga is technologically challenging” or “I feel socially connected when I practice yoga online”. Results will report agreement with individual statements, as the scores had low reliability as a composite scale (α = .49).

*Yoga practice and dosage*: several questions assessed the medium and frequency of yoga practised prior to and during lockdown (self-led, in-person classes, recorded video, live-stream, etc.), online yoga platforms used, years of practice and reasons for not continuing yoga since the pandemic. Dosage was measured by hours per week (HPW) and days per week (DPW) of yoga practice. Instructors were asked additional questions about their teaching of online yoga.

Qualitative data relating to experiences of practising yoga during the lockdown were elicited through several open-ended questions, including motivations for practising and specific experiences of online yoga (“what you liked and what you found challenging or disliked”). Yoga instructors were additionally asked about their experiences of teaching online yoga.

### Analysis

Quantitative data was analysed using SPSS version 26, and the alpha level was set to p < 0.05. Descriptive statistics were calculated for socio-demographic characteristics, yoga practice and psychosocial health variables, for both teachers and practitioners. Median dosage HPW was used to create a dichotomous variable for high (≥ 3 students, ≥ 5 teachers) and low dosage (≤2 students, ≤ 4 teachers). A bivariate correlation matrix was conducted to explore significant correlations between yoga variables and psychosocial health scores. Hierarchical linear regression was used to assess the association between age, gender, yoga practice, living situation, employment status, and psychological outcomes (stress, depression, anxiety, and social connectedness). The model’s explanatory power was indicated by the change in R-squared and ANOVA results.

Open-ended responses relating to online yoga experiences were analysed inductively using descriptive thematic analysis within a realistic framework [62]. Qualitative research offers greater insight into participants’ experiences, providing a rich complement to the quantitative methods in this study . Consistent with this epistemological stance, coding was conducted at a semantic level, focusing on the explicit content of responses rather than underlying assumptions or interpretations. This approach is suited to descriptive analyses of short, open-ended survey responses. Responses were downloaded directly from Qualtrics into Excel spreadsheets for practitioners and teachers separately; any typographical errors were corrected. During the initial reading, responses were checked for relevance and divided into benefits and barriers in relation to experiences of online yoga. Responses were read multiple times by LM to produce a list of codes and emergent themes representing recurrent patterns in the data, which were then reviewed and discussed with an experienced qualitative health researcher (TC). Initial analysis focused on practitioners’ responses, with instructors’ reflections on practising and teaching online yoga subsequently integrated, leading to iterative development of the thematic content and structure through multiple discussions amongst the research team. The number of responses relating to each theme (n) is reported to indicate prevalence.

## Results

Of the 1295 participants who responded to the survey, 1185 met the inclusion criteria (511 practitioners, 586 teachers). For those who identified as yoga teachers, 20.3% were full time, 69.4% part time and 10.3% certified but not currently teaching. Teachers ranged in age from 22 to 76 years (M = 48.78, SD = 11.19), were mostly female (94.9%), Caucasian (87.9%), and well-educated (75.4% had a bachelor’s degree or higher). Yoga students ranged in age from 18 to 80 years (M = 45.26, SD = 14.48), were mostly female (92.8%), Caucasian (88.6%) and held a bachelor’s degree or higher (78%), Table 1.

**Table 1:** Socio-demographics characteristics of sample.

Variables	All, n* (%)	Students, *n* (%)	Teachers, *n* (%)
**Gender**	1184	511	585
Male	71 (6)	34 (6.7)	30 (5.1)
Female	1109 (93.7)	474 (92.8)	555 (94.9)
Other	4 (.3)	3 (.6)	0 (0)
**Ethnicity**	1184	511	585
Asian/Asian American/Asian British	53 (4.5)	18 (3.5)	30 (5.1)
Black/Black **British/African American**	12 (1)	4 (.8)	8 (1.4)
Hispanic/Latino	16 (1.4)	12 (2.3)	4 (.7)
White	1043 (88.1)	453 (88.6)	514 (87.9)
Other	60 (5)	24 (4.7)	29 (5)
**Employment (during lockdown)**	1183	585	511
Employed	727 (61.5)	282 (55.2)	397 (67.9)
Unemployed	270 (22.8)	171 (33.5)	74 (12.6)
Furloughed (lost job due to Covid-19)	150 (12.7)	43 (8.4)	98 (16.8)
Other	36 (3.1)	15 (3)	16 (2.7)
**Education**	1181	510	583
GCSE, Secondary school	77 (6.5)	38 (7.5)	31 (5.3)
GED, A levels	136 (11.5)	50 (9.8)	74 (12.7)
Associates Degree	64 (5.4)	24 (4.7)	38 (6.5)
Bachelor’s Degree	440 (37.3)	201 (39.4)	204 (35)
Master’s Degree	367 (31.1)	150 (29.4)	188 (32.2)
Doctorate	97 (8.2)	47 (9.2)	48 (8.2)
**Location**	1020	437	513
United Kingdom	630 (61.8)	295 (67.5)	292 (56.9)
United States	236 (23.1)	98 (22.4)	119 (23.2)
Ireland	26 (2.5)	4 (.9)	20 (3.9)
Canada	20 (2)	7 (1.6)	12 (2.3)
Finland	13 (1.3)	2 (.5)	11 (2.1)
Sweden	12 (1.2)	3 (.7)	9 (1.8)
Australia	11 (1.1)	3 (.7)	8 (1.6)
India	10 (1)	2 (.5)	7 (1.4)
Other Countries (<10 participants each)	62 (19.4)	24 (5.5)	35 (6.8)
**General Health Status**	1185	511	586
Excellent	249 (21)	87 (17)	149 (25.4)
Very good	358 (45.4)	233 (45.6)	272 (46.4)
Good	335 (28.3)	154 (30.1)	148 (25.3)
Fair	55 (4.6)	33 (6.5)	17 (2.9)
Poor	8 (.7)	4 (.8)	0 (0)
**Living Situation (during lockdown)**	1184	511	586
Alone	156 (13.2)	52 (10.2)	88 (15)
w/ Partner/spouse	443 (37.4)	195 (38.2)	227 (38.7)
w/ Partner and child(ren)	375 (31.7)	145 (28.4)	202 (34.5)
w/ Child(ren)	53 (4.5)	23 (4.5)	25 (4.3)
w/ Parent(s)	43 (3.6)	24 (4.7)	15 (2.6)
w/ Parent(s) and sibling(s)	36 (3)	26 (5.1)	5 (.9)
Other family	19 (1.6)	11 (2.2)	7 (1.2)
w/ Roommate(s)	59 (5)	35 (6.8)	17 (2.9)
**Type of Location (during lockdown)**	1181	510	584
Urban	331 (26.3)	134 (26.3)	156 (26.7)
Suburban	572 (48.4)	263 (51.6)	262 (44.9)
Rural	298 (25.2)	113 (22.2)	166 (28.4)

*Includes those who did not identify as teacher or student.

The majority of participants reported they were spending about the same amount (*n* = 405, 35.2%) or more time (*n* = 494, 43%) practising yoga during the pandemic. Participants reported self-led practice (59.8%), online recorded classes (47.3%), and live-stream classes (53.5%). Only 3.5% reported not practising yoga during this period. When asked about using online yoga post-lockdown, participants reported they would probably or definitely continue to take recorded online classes (46.1%) or live-stream classes (45%). The majority of participants used Zoom, Facebook and YouTube to access online classes.

### Psychosocial health

Overall, participants had normal mean scores for depression, stress and anxiety, and were experiencing some isolation, as shown in Table 2. Using established cut off scores, 80.3%, 82.3%, and 85.9% of participants had normal/mild scores on depression, anxiety, and stress subscales, respectively. 19.7% of participants exhibited some symptoms of depression (moderate, 11.6%; severe, 4.2%: extremely severe 3.9%), 17.7% exhibited anxiety (moderate, 11.1%; severe, 3.2%; extremely severe, 3.4%), and 14.0% exhibited stress (moderate, 7.8%; severe, 4.5%; Extremely severe, 1.7%).

**Table 2:** Psychosocial well-being and yoga variables.

Variable	All* Mean (± *SD*)	Students	Teachers
Depression	8.41 (±7.49)	8.97 (±7.45)	7.91 (±7.5)
Anxiety	4.64 (±5.77)	5.08 (±6.03)	4.26 (±5.52)
Stress	11.02 (±7.82)	11.7 (±7.87)	10.43 (±7.74)
Social Connectedness (HFS)	18.04 (±3.59)	18.17 (±3.61)	17.92 (±3.57)
Hours per week (HPW)*	5.69 (±3.58)	4.28 (±2.46)	7.03 (±3.93)
Days per week (DPW)*	4.00 (±2.05)	3.16 (±2.03)	4.72 (±1.77)
Years of yoga practice	12.33 (±10.31)	8.15 (±8.91)	15.99 (±10.07)
Barriers of yoga	1.78 (±.68)	1.92 (±.69)	1.60 (±.59)
Benefits of yoga	4.54 (±.50)	4.49 (±.53)	4.60 (±.44)

Depression: normal (0–9), mild (10 –13 ), moderate (14 –20 ), severe (21 –27), extremely severe (28+). Anxiety: normal (0–7), mild (8 –9 ), moderate (10 –14), severe (15 –19), extremely severe (20+). Stress: normal (0–14), mild (15 –18), moderate (19 –25), severe (26 –33), extremely severe (34+). Social Connectedness: very isolated (0–11), isolated (12 –15), some isolation (16 –18), socially connected (19 –21), very socially connected (22 –24 ). ** Dosage questions pertain to yoga practice at home during lockdown months only

For teachers, DPW correlated significantly with stress (*r* = −.152, *p* < .001), depression (*r* = −.142, *p* < .001), social connectedness (*r* = .111, *p* < .001), and perceived barriers to yoga (*r* = −.203, *p* < .001). For students, DPW correlated significantly with stress (*r* = −.100, *p* < .001), depression (*r* = −.138, *p* < .001), perceived barriers (*r* = −.343, *p* < .001) and benefits (*r* = .187, *p* < .001) of yoga. See S1 Table for the full correlation matrix of psychosocial health and yoga variables, including HPW, which had similar significant relationships to psychosocial health. Years of practice was significantly correlated with stress (*r* = −.205, *p* < .001), depression (*r* = −.138, *p* < .001), anxiety (*r* = −.193, *p* < .001), and social connectedness (*r* = .064, *p* < .05).

Hierarchical regression models with age and gender entered in the first step and yoga variables, living situation and employment status in the subsequent steps were significant in predicting criterion variables, but had low predictive power (See S2 Table). Controlling for age and gender, frequency of yoga practice (DPW) was associated with lower depression, whilst years of practice was associated with lower anxiety. Living situation (not alone) and being employed were associated with greater social connectedness, while living alone and being unemployed were associated with higher depression. Age was inversely associated with depression, anxiety, and stress.

### Perceptions and experiences of online yoga

**Fig 1:**
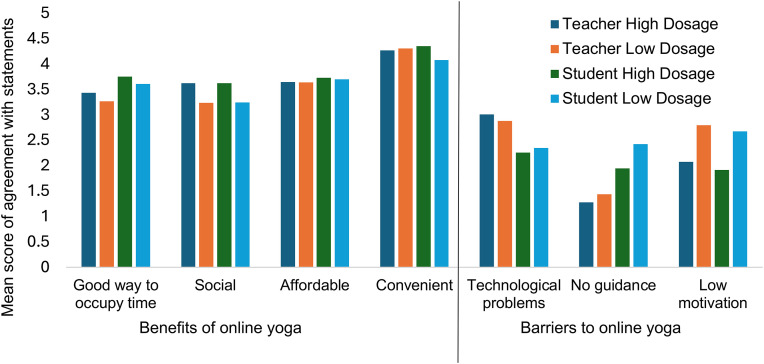
Perceived benefits and barriers of online yoga.

Participants agreed more strongly with statements relating to the benefits of online yoga compared with the barriers, as shown in Fig 1. The most positively endorsed benefit of online yoga was convenience. Technological issues were most prominent in terms of barriers. Students with lower dosage perceived a lack of personal guidance to be a greater barrier to online yoga, while low dosage teachers and students struggled with motivation in practising online yoga.

Qualitative findings expanded our understanding of both student and teacher experiences of online yoga. Analysis of open-ended responses produced eight themes (four benefits and four barriers), as seen in Tables 3 and 4 (with number of responses). All quotes are in italics, followed by the participant’s gender (M = male, F = female) and age.

**Table 3:** Benefits of Online Yoga (n = 591): Themes, subthemes, and indicative quotes.

Bolstered community spirit	(Students, n = 147; Teachers n = 336)
Familiar teachers	“One of the online instructors I am familiar with and made me feel like I was back at a regular class” – F, 62 “It helped that I’m continuing classes with my regular teacher, so it’s a familiar set of poses and I’m used to the language that she uses” – F, 49 “My health and how I feel is my primary motivator. On Wednesday morning I take my class with my teacher, I do look forward to seeing her since we are not able to meet in person right now” – F,61 (Teacher)
Seeing other students	“Nice sense of community with like-minded people during a hard time” – F, 20 “Opportunity for social interaction, having a chat with fellow students” – M, 66 “Checking in with my yoga teacher. Keeping up a regular practice. A shared sense of community with other practitioners” – F,40 (Teacher)
Togetherness	“Knowing that so many people are practicing together at home has been very motivating” – F, 46 “It is amazing being able to practice knowing other people are doing it too at the same time – togetherness” – F, 45 “To keep a connection with my long-term regular teacher and my fellow students. To continue to get new ideas for practising and teaching (once I return to that). For my own physical and mental well-being and spiritual growth.”- F,39 (Teacher)
Convenience	n = 133 (Students); n = 207 (Teachers)
Accessibility	“I like being able to attend classes that are usually too far away, not having to travel after savasana, being able to still do my classes” F, 50 “No travelling involved means I can attend my favourite yoga teacher’s classes even though she has moved to a different area” F, 57 “No room hire or other overheads, Able to do it from anywhere with WiFi., No space limitations. Able to offer more classes and at different times as commute is not an issue. Able to have students from outside my city. Shy students can remain anonymous and still do the practice from the comfort of their home.” F, 46 (Teacher) “I charge less so some students come more often. Less to organise as I do not have to travel” F, 58 (Teacher)
Abundance of choices	“I like the variety of choice that I can pick and choose different types depending on my mood and energy levels” – F, 43 “There is plenty of choice online. I have the option to choose a short or longer session to fit in with my schedule, find targeted routines” – F, 58 “I can practice with different teachers and students all around the world, the offer seams endless” F, 50 (Teacher)
“It is a lifeline”	n = 124 (Students); n = 234 (Teachers)
Mental health benefits	“Being in lockdown is quite stressful mentally and I have found online yoga classes essential to keep me mentally healthy” F, 54 “Grateful to have the time to practice more than I did prior to the lockdown. It helps me to regulate during this time, gain perspective, and navigate through the unknown and stress of this time period (F, 55). “At the start of lockdown it was a lifeline to help me deal with anxiety and worry for the future. It helped me sleep and see things more clearly. It continues to be a lifeline of support in my life” F, 41 (Teacher)
Physical health benefits	“Not only has my chronic pain decreased more in the past 30 days than it has in the past 9 months, but it has also helped provide a great source of meditation that I didn’t even know I needed…I’ve also become more flexible, less stressed, stronger and more toned” F, 23 “I have noticed physical improvements, I have noticeably toned up more since lockdown” F, 41 “I am a yoga teacher! So need to for my work. But aside from that, gives me mental respite and physical health benefits” F,33 (Teacher)
Enjoyable	“Enjoyed being able to continue practicing yoga” M, 53 “For general wellbeing. I enjoy it. I enjoy learning from other experienced teachers. For savasana!” F, 30 (Teacher) “Loved being in my own space whilst still connected to a live teaching. F, 50 (Teacher)
Developing personal practice	n = 47 (Students); n = 51 (Teachers)
Non-competitive	“I can concentrate on myself and not compare myself to others in class” F, 61 “I enjoy online yoga as you are not distracted by other people where there is a tendency to compare and compete with others. The practice becomes more internal and less external” F, 54 “Students are safe at home without opportunities to compare their practice to others and so can withdraw easier.” F, 55 (Teacher)
Doing my own thing	“If there are any poses I feel called to do that weren’t covered in the class I can do them on my own” F, 27 “I find benefit from the ability to pause and rewinding through pre-recorded sessions as I can approach the class at my own pace” M, 23 I feel a freedom to try new things at home in my practice F, 25 (Teacher)

**Table 4:** Barriers to online yoga (n = 490): Themes, subthemes, and indicative quotes.

Lack of group energy	n = 87 (Students); n = 153 (Teachers)
Lacking motivation	“I love the connection of practicing in a room with other people, as it motivates and inspires me” F, 27 “I miss the encouragement you get from practicing with a group. I find it’s easier to slack when I am at home alone” F, 26
Lack of social interaction	“I prefer in-person classes so I can get bodily adjustments and have social interactions with the teacher and classmates” F, 36 “It’s tough to get the same vibe with online yoga. Miss the people and the energy!” F, 23 “A more impersonal affair, cannot enjoy the small things such as a chat with students individually or even appreciating the journey to classes as all teaching at home” F, 28 (Teacher)
No group vibes	“It doesn’t replace the real experience as you have to imagine that the group energy is there rather than experience it which is way more powerful” F, 65 “The students and I miss the energy, the sense of others breathing in the room, the social connection and proximity of others” F, 57 (Teacher)
Lack of personalised guidance	n = 75 (Students); n = 129 (Teachers)
No hands-on adjustments	“I miss the tutor being able to give specific alignment cues and physically touching me” F, 41 “I miss the physical, hands-on adjustments” F, 71 “Not able to provide hands on adjustments or help students with placement of props” F, 60 (Teacher)
No individual feedback	“I worry about not receiving active guidance from the teacher to correct or improve my form” F, 27 “Instructors tend not to give feedback” F, 60 “I cannot offer individual attention to students, cannot do any practices that require teacher supervision, cannot introduce new techniques” F,44 (Teacher)
Feeling unsafe	“I dislike the fact that if I’m doing a pose incorrectly there’s no one there to help me” F, 39 “I dislike the lack of posture realignment, I could be doing stuff really badly” F, 55 “Cannot see the students’ body for safety and alignment. Unless a student is familiar with the online teacher prior to this quarantine, I feel students cannot safely follow the practice” F, 63 (Teacher)
Technological issues	n = 61 (Students); n = 104 (Teachers)
Connection/ equipment	When taking live-stream classes I found it sometimes frustrating as the screen would freeze” F, 60 “Problems with connection could cause interruption to sound and vision meaning you couldn’t follow the class” F, 61 “The faffing about gooseneck device-holder and laptop. The loss of internet connection during a class!” F, 39 (Teacher)
Small screens	“I am on an iPad so screen can be small but I manage. Sometimes I have to put my glasses on to see a pose on the screen” F, 64 “It was hard to understand what to do from the small Zoom window, even though I mirror screened to my big TV” F, 62 “I can’t see everyone clearly as there are several on a small screen so never sure how people are finding it and can’t adapt the class as easily to the ‘vibe’ in the room” F, 32 (Teacher)
Inadequate Ambiance	n = 58 (Students); n = 21 (Teachers)
Environment	“I don’t own the accessories or have the best ambiance for this practice so it loses some of the reasons I loved going to my studio classes” F, 53 “I have disliked needing to use cramped space at home as it has limited the moves I can do” F, 47 “Hard to find space and peace at home” – F, 55 “Atmosphere not the same. Difficult working space for me at home” F, 60 (Teacher) “Teaching online in general is much more tiring than teaching in person. I also cannot control the environment – no incense, no music, no scene setting and very few props. None of that really matters but they are nice rituals and add depth and personality to a class” F, 45 (Teacher)
Temperature	“I miss the warm studio with underfloor heating, enabling hot yoga classes to be offered” F, 50 “Cooler than hot yoga studio which took about four sessions to get used to differences in practicing in cooler temperatures at home” F, 54
Distractions	“It’s hard for me to tune out all the distractions while practicing at home” F, 37 “Certainly is harder to get the same level of workout when not in the hot room and have noise of kids and pets walking through” F, 49 “Less interaction, less students and distractions by family members” F, 53 (Teacher)

### Benefits of online yoga

Bolstered community spirit was the most prevalent theme, characterised by feelings of social connectedness from practising with others. Secondly, the convenience of online yoga was highly valued, through increased accessibility and abundance of class choice for all levels of experience and capability. The third theme, “it is a lifeline”, encapsulates the mental and physical health benefits, including mitigations to the negative impact of the pandemic. The fourth theme related to the unique value of online yoga in developing a personal practice, free from social comparison (see Table 3).

### Bolstered community spirit

A prominent benefit of streamed online yoga was an appreciation of the overall “togetherness” felt when practising in a group, particularly when led by a familiar instructor. Students reported a feeling of ease, comfort and normality that came with continued practice with regular teachers. They also enjoyed seeing familiar yoga friends and connecting with like-minded people from around the world. One participant reflected that she “quickly felt part of a community. Easy and energetic chat before class made it feel like we were at our usual studio” (F, 60). The prevalence of this theme may be specific to lockdown circumstances, given the restrictions to in-person social interaction during this period. However, connecting with others may also have benefits beyond socialization, impacting on overall engagement and depth of practice: “My experience is enriched when the instructor knows me or says hello when my name appears on the screen. Overall, much prefer live as I’m likely to push myself more and stay focused” (F, 42).

Indeed, the instructor’s ability to personalize the practice and build group connectedness increased students’ motivation. Participants discussed feeling a sense of accountability in live-stream classes compared with recorded classes, with a dedicated “space” and time to practice with others. Teachers reflected on the value of connection with other teachers as well as students, having the opportunity to learn from different teachers and “supporting the yoga industry in difficult times” (F,37).

### Convenience

As seen in the quantitative findings, the convenience and accessibility of online yoga was a dominant theme for both students and teachers (“people can roll out of bed to their mat” F, 45). Students discussed the ease and cost-effectiveness of practicing at home. Removing the need to travel saved time, money, and the challenge of finding childcare, with the majority of online classes also cheaper than in-person classes. The removal of geographical barriers enabled access to a wider range of yoga teachers, with participants reporting their enjoyment of re-joining previous teachers who had moved, or practising with well-known instructors across the world (“I am so grateful to have my gurus in my living room”, F, 37). The wide range of class choices in terms of length, style, and time of class made online yoga more easily fit into personal schedules and facilitated more frequent practice for many participants. This abundance of choice was especially relevant for those using recorded classes on YouTube or a subscription website. Teachers also enumerated the additional benefits of low costs for themselves and students, low overheads and high scalability, despite the majority (70%) also reporting they earned less compared to pre-pandemic.

### It is a lifeline

Participants reported multiple benefits of practicing online, for mental and physical health and broader wellbeing, consistent with benefits attributed to yoga more generally. Participants also reflected on online yoga as an important buffer against the specific stressors and challenges of life during the pandemic. It became a time to look forward to being with friends online or practising together with family members in the house: “How cool is it that my sister and I get to hang out while dually improving our bodies and minds?” (F, 23). Many participants discussed feeling intrinsically motivated to practice online, reflecting that online yoga classes were “enjoyable” and even essential during this period: “It is a lifeline. My yoga teachers are exceptional and created a safe, fun and challenging environment that we as individuals could continue our practice under their experience and watchful eye” (F, 60). Teachers recounted the benefits of having ample time to deepen their own practice, offer additional support to their students and a wider range of classes: “…now I can offer more emotional support, as we get to chat before and after, I can offer more than before, as we are not stuck in a studio’s schedule” (F, 41).

### Developing a personal practice

Whilst a minor theme, the ability to develop a personal practice through online delivery, free from competition and distraction highlights a unique benefit of online delivery and potential barrier of in-person yoga for some individuals. For these participants, the non-competitive and non-judgemental nature of online yoga allowed them to feel more comfortable trying new poses, going at a slower pace, or stopping and rewinding when needed (in recorded classes). For participants new to yoga, online yoga classes were regarded as “less intimidating”. For more seasoned participants, the decrease in competitiveness allowed for a deeper, internalised, and more personal practice.

### Barriers to online yoga

Whilst a sense of community was perceived as a key benefit of online yoga, for others, perceived lack of ‘group energy’ negatively impacted motivation and engagement, see Table 4. In the second theme, students and instructors shared concerns about the lack of personalised guidance to ensure student safety. Unsurprisingly, technological issues were highlighted as barriers to the online experience. The fourth theme related to challenges in creating an appropriate ambience to support home practice, through lack of space, equipment and multiple distractions in the home environment.

### Lack of group energy

The most frequently mentioned disadvantage of online yoga was that the “group energy is missing” (F, 44) compared with in-person classes. Participants discussed the lack of social interaction as a shortcoming of online yoga, highlighting the value placed on social connection in creating a supportive yoga environment and “experience”: I miss the camaraderie of doing the classes in person. I miss the social togetherness of everyone being in the same room and the sense of community being with other people brings (F, 54).

This lack of “vibe” and “group energy” impacted on motivation, both in terms of attendance (“It’s difficult to motivate myself to get on the mat alone”, F, 53) and engagement (“Feels generic and impersonal. Don’t work as hard”). This contrasts with participants who valued the privacy and non-competitiveness of online classes. Similarly, teachers expressed dissatisfaction with the lack of bonding with the class and inability to gauge the needs of the class: “Missing the personal interaction. Harder to grasp the rhythm and energy of the class…” (F,46).

### Lack of personalised guidance

Despite taking live classes with familiar teachers, for some, the lack of personalised guidance impacted on their enjoyment. Students reported missing individualised attention in order to improve and develop their practice as well as expressing concerns about safety. In particular, less experienced participants reported apprehension about trying unfamiliar poses without a teacher’s full guidance. Experienced and new students alike missed the verbal and physical adjustments that yoga teachers typically provide in classes.

Challenges arose regarding how to offer and receive instructor feedback. Whilst some students desired individual feedback, expressing dissatisfaction with blanket statements of encouragement directed at the whole group, others found it embarrassing to be corrected publicly. Personal feedback could also be interpreted as divisive or simply distracting in a live-stream group class: “A teacher has started metering out praise or comments for individual students in a zoom class, which I find irritating as it is distracting” (F, 58). Communication challenges and lack of hands-on adjustment were similarly discussed by teachers, with safety concerns most prevalent. This could limit the style of yoga and type of practice offered online.

### Technological issues

Participants commonly reported frustration at poor Wi-Fi connection, sound quality, and the inconvenience of looking at a small screen. Due to the dynamic nature of most yoga classes, participants expressed frustration of the need to move their device so the teacher could see them in different postures. The continuity of the class could also be interrupted by a poor connection. Additionally, some participants felt they did not have the proper equipment and that not all teachers had high-quality microphones and lighting. Teachers recalled the difficulty in seeing their students properly due to small screens, technical issues, and inability to play music: “I have only a small screen to watch all students. Often I cannot see a student’s asana, so I cannot give proper feedback” (F,44).

### Inadequate ambiance

Whilst participants highlighted the convenience of online yoga and many enjoyed practising in the home, some students struggled to find an appropriate environment to practice. This could refer to a lack of appropriate physical space or missing the “atmosphere” and facilities of a studio. Several students noted that they missed attending their studio specifically for hot yoga. Both students and teachers both expressed a need for a space free from distractions, such as family, pets, phones, and other obligations: “I feel like I’m having a hard time really getting into it and being mindful because I’m in a non yoga atmosphere at home. I long for the safe, calm and quiet haven of a studio once again!” (F, 35). Some teachers also noted that space, noise and distractions in their home limited their capacity to offer classes, whilst lack of control over the online ambience and need to mute students during practice made classes less friendly.

## Discussion

This large cross-sectional survey provided unique insight into the mental health and stress levels of yoga practitioners during the first wave of the COVID-19 pandemic, when global surveys showed a significant spike in mental health problems [27,28,63]. In contrast, this study revealed low levels of stress, anxiety and depression symptoms amongst yoga practitioners during this period. Furthermore, higher yoga dosage and years of practising were related significantly, if weakly, to favourable mental health. Using a mixed methods approach, the study also contributed to our understanding of the benefits and challenges associated with online delivery, from the perspective of both teachers and students. Consistent with intervention-based studies, we found that online yoga offered practitioners an important way to connect with others and was particularly valued for its benefits for mental wellbeing. Greater access to a wider range of teachers and styles of yoga, alongside the convenience and reduced cost of online yoga increased accessibility and practice of yoga. Barriers largely related to practical challenges around technology, home environment, and lack of personalised guidance and group connection. Interpreted within the framework of the COM-B model, this offers important insights for the design and implementation of yoga interventions utilising online or hybrid delivery models in the post-pandemic era.

Our qualitative findings indicated that online yoga provided a valued resource to help manage the stress, uncertainty and social isolation arising from the COVID-19 pandemic. During the initial phase of the pandemic, global surveys and reviews recorded deteriorations in population mental health and well-being, particularly amongst women [28,63]. Whilst we were not able to make comparisons with baseline data, our quantitative findings indicated that the majority of our sample had normal levels of depression, anxiety and stress, based on established cut-off scores, with lower levels of negative affect than population surveys during this period [64,65]. Additionally, the frequency and duration of yoga practice were associated with better mental health. Whilst causality cannot be inferred from our correlational findings, this aligns with broader research supporting the positive impact of yoga on negative affect, depression, anxiety and stress [11,14,12,66]. Importantly, our qualitative findings provide further insights into the impact of yoga during this period on mental and physical health, as well as suggesting key mechanisms.

Consistent with previous findings with both online and in-person classes, yoga was seen as a ‘lifeline’ for many during the pandemic, providing tools to manage psychological distress, worry and anxiety [30], with 43% of our sample reporting increases in their yoga practice. Socio-emotional support was provided by feeling part of a yoga community, and connecting with other students, family members and familiar teachers. The benefits of yoga for increasing social connectedness have been reported previously with in-person studies [25,67], and it is encouraging that this can be successfully translated into online delivery with the help of skilled teachers [40,47]. Physical health benefits included increased strength and tone, flexibility, fitness, management of pain and sleep [16]. While the value placed on the social benefits of online yoga may have been specific to the pandemic circumstances, it supports the potential of tele-yoga to increase access to benefit rural and socially isolated populations [32,48,68].

The acceptability of online delivery of yoga in this population-based study aligns with clinical and intervention studies [32]. Participants were often surprised at the quality and breadth of online offerings, whilst also acknowledging that the experience is “not the same” as in-person classes. The majority of teachers also reflected positively about transitioning to online teaching, at least in the context of the pandemic restrictions, with some acknowledging their role in supporting the yoga community during such a challenging time. Principal disadvantages related to the reduced social interaction and connection of online delivery, along with concerns around not being able to see students sufficiently. Online delivery was viewed as a ‘compromise’, convenient but less enjoyable to teach. The majority of participants reported that they would continue with online yoga after the lifting of pandemic restrictions, reflecting on the benefits of increased convenience and access as noted elsewhere during COVID-19 [47,68] and in studies with clinical populations [66]. Future studies are needed to evaluate the current usage and experiences of online versus in-person yoga classes.

One of the key aims of the study was to better understand the facilitators and barriers to online yoga delivery using the framework of the COM-B model [49], in order to feed into the design and implementation of online yoga provision and future interventions. Capability (knowledge, skills and abilities) was enhanced by the wide range of online yoga classes catering for all levels of experience and scheduling. Live stream classes supported accountability, consistent with previous findings [40,41], and when well matched with ability instilled a sense of physical competence. Recorded classes offered greater flexibility and enabled individuals to develop and deepen their personal practice at their own pace, increasing confidence. In contrast, a key barrier reported by both teachers and students were concerns around safety and lack of personalised adjustments and guidance, as highlighted in several previous studies [42,48,68]. This was particularly salient for less experienced practitioners and some teachers reflected on the need to modify class content given the difficulty of viewing students’ alignment and ‘reading the room’ to respond to student needs. Such challenges may be mitigated by small class sizes, familiarity with teachers/student cohorts and hybrid models of teaching [41,47]. Technological issues unsurprisingly dominated both quantitative and qualitative responses, but can potentially be offset by strong motivation for “high value activities” [42] as described below.

Opportunity relates to physical, environmental and social factors that enable yoga practice. As expected, the convenience and affordability of online classes were widely cited as a primary benefit of online yoga [32,47,68], enabling participants to maintain or increase their yoga practice. Indeed, more than two-thirds of participants reported practising as much or more frequently than previously. Participants were grateful to have access to more classes, at a variety of times, styles and durations with international teachers. Having the opportunity to socially interact and share the practice with others, including friends and family, was also highly valued. Whilst it is unclear whether these benefits would be sustained post-pandemic, the convenience of online yoga does overcome traditional barriers of access to yoga, such as class availability, affordability and clashes with family and work demands [6,53]. Some studies have also found higher adherence with at-home or online yoga interventions [35,40,47]. However, limited space, lack of equipment and multiple distractions within the home environment impacted enjoyment and ability to practice yoga. This is particularly salient when considering the importance of reducing health disparities – tele-yoga potentially increases access through flexible, affordable yoga provision but paradoxically, uptake may be limited by a lack of physical space in more deprived areas [6,32].

Finally, autonomous and reflective motivation influences the likelihood of behaviour uptake and maintenance [49]. Consistent with previous findings [3,4,58], we found that the mental and physical health benefits of yoga were strong reflective motivations for practising online yoga, including managing pandemic stressors. Enjoyment of yoga provided strong intrinsic motivation, although some reflected that practising online reduced accountability, which reduced their motivation and effort invested. A sense of social connectedness enhanced class enjoyment, but its absence negatively affected engagement and motivation. Based on our findings and previous research, instructors played a crucial role in fostering an online setting that promoted a sense of community and support [41,42,47]. Indeed, some teachers noted the potential to offer greater emotional support to students outside the constraints of studio teaching. The value of meeting relatedness needs may have been particularly salient given the context of COVID-19 restrictions, in line with self-determination theory [69].

Strengths of the study include a large community sample incorporating perceptions of both practitioners and teachers of online yoga. A mixed methods approach enabled the triangulation of quantitative and qualitative findings, enabling a more comprehensive understanding of the potential mechanisms through which online yoga impacts on health. Additionally, the application of a theoretical framework to understand barriers and facilitators to effective implementation of online yoga enhances transferability, providing valuable insights for future online and hybrid yoga interventions.

Several limitations should, however, be acknowledged. As with previous surveys, our sample underrepresented men, racial and ethnic minority groups, and individuals without university education, thus limiting the generalisability of the findings. Additionally, recruitment and participation in online yoga necessitated access to technology, introducing a bias towards the digitally literate. Nevertheless, we captured a wide age range of participants engaging with online yoga. However, lack of digital literacy or access may reinforce health inequalities, so access and technological training should be considered to address these barriers when delivering online yoga in an intervention context. Future studies should adopt strategies that enhance representativeness and inclusion, such as targeted social media recruitment, culturally relevant promotional materials, and collaboration with community organisations to engage more diverse yoga practitioners. The cross-sectional design precludes causal inferences about the relationship between yoga and psychosocial health; observed associations should be interpreted as correlational only. Additionally, the newly developed measure of benefits and barriers of online yoga, showed low internal reliability, limiting interpretation beyond the individual statements. Finally, as the study was conducted during the COVID-19 pandemic, when physical restrictions were in place, this is likely to have shaped evaluations of online delivery. This highlights the importance of future research to explore perceptions and experiences of online yoga in a post-pandemic setting.

Our findings have direct relevance for practice, particularly in terms of designing yoga interventions which successfully incorporate an online component in their delivery. The widescale uptake of online yoga during the pandemic demonstrates that tele-yoga can increase access and uptake through reducing traditional barriers such as cost, travel and availability, with potential to increase adherence and home practice. In terms of delivery medium, live-stream classes are most effective to enhance engagement, accountability and social connectedness. For novice practitioners, recorded sessions support skill development and self-efficacy by allowing individuals to work at their own pace and build their practice. Hybrid models that incorporate both synchronous (whether in-person or online) and asynchronous delivery may therefore be the most effective intervention model to establish a consistent yoga practice of sufficient dosage to produce beneficial health outcomes. Combining in-person sessions with online delivery may offer a sustainable hybrid model that optimises engagement and social connection whilst retaining the enhanced accessibility that online delivery provides. Based on the current findings and broader literature, considerations and recommendations for optimal online yoga delivery and implementation are summarised in Table 5.

**Table 5:** Framework for optimising online yoga delivery using the COM-B model.

COM-B construct	Barriers/facilitators	Strategies
Capability	• Safety concerns for novice practitioners. • Lack of individualized guidance. • Technological issues.	• Offer smaller, beginner-orientated classes; provide an initial 1-to-1 session and ongoing support outside classes. • Build confidence and competence through clear, personalized verbal instruction, with emphasis on body-awareness; offer supplementary recorded classes to build self-efficacy and home self-practice. • Assess digital literacy in target population and tailor onboarding/training accordingly.
Opportunity	• Increasing access to wider demographic. • Affordability and flexibility of classes. • Access to appropriate home environment.	• Work with community organizations to reduce access barriers and highlight meaningful population-specific benefits. • Provide access to low-cost or free classes, and flexible scheduling (e.g., evenings or shorter sessions). • Offer modified practices (e.g., chair yoga, use of household props) to support practice.
Motivation	• Desire for community and social connection • Awareness of health benefits. • Enjoyment and accountability	• Foster an online environment that feels safe, supportive, and connected; include time for social interaction before/after classes. • Emphasize mental and physical benefits of yoga, even when practised virtually. • Include synchronous classes and interactive elements to increase engagement and accountability.

## Conclusions

This study uniquely examined the experiences of practising online yoga during the COVID-19 pandemic from the perspective of both yoga teachers and practitioners. The mixed methods approach provided insight into the psychosocial health of practitioners during this period and suggested mechanisms of impact. Importantly, it highlighted key barriers and facilitators to the effectiveness of tele-yoga in a large community sample, providing a framework for the design and implementation of future yoga interventions utilising online or hybrid delivery models. Whilst tele-yoga has potential for increasing access to yoga, it is essential that future research continues to explore how to make online yoga interventions meet the needs of under-represented populations.

## Supporting information

S1 Table Correlations. (DOCX)

S2 Table Results of linear regression models predicting psychosocial variables from demographic, yoga practice variables, and living situation and employment. (DOCX)
