# Supplementary material for: ‘It is a lifeline’: International cross-sectional survey of benefits, barriers and acceptability of online yoga during the COVID-19 pandemic
Source: PLoS One. 2026 Feb 18;21(2):e0341852. doi: 10.1371/journal.pone.0341852 (PMC12915962; doi:10.1371/journal.pone.0341852)
Supplement: S1 Table — (DOCX) [file pone.0341852.s001.docx]

**S1 Table. Correlations**

Table A: Correlations between yoga dosage, psychosocial variables and perceived benefits and barriers to yoga for yoga teachers

|  | DPW | HPW | Stress | Anxiety | Depression | Connectedness | Yoga Benefits |
| --- | --- | --- | --- | --- | --- | --- | --- |
| Days per week (DPW) |  |  |  |  |  |  |  |
| Hours per week (HPW) | .589** |  |  |  |  |  |  |
| Stress | -.152** | -.145** |  |  |  |  |  |
| Anxiety | -.076 | -.060 | .635** |  |  |  |  |
| Depression | -.142** | -.135** | .573** | .545** |  |  |  |
| Connectedness | .111** | .027 | -.221** | -.194** | -.369** |  |  |
| Yoga Benefits | .031 | -.011 | .065 | .032 | -.057 | .089* |  |
| Yoga Barriers | -.203** | -.143** | .237** | .227** | .280** | -.205** | -.205** |

|  | DPW | HPW | Stress | Anxiety | Depression | Connectedness | Yoga Benefits |
| --- | --- | --- | --- | --- | --- | --- | --- |
| Days per week (DPW) |  |  |  |  |  |  |  |
| Hours per week (HPW) | .706** |  |  |  |  |  |  |
| Stress | -.100* | -.124** |  |  |  |  |  |
| Anxiety | -.043 | -.081 | .691** |  |  |  |  |
| Depression | -.138** | -.124** | .689** | .622** |  |  |  |
| Connectedness | .067 | .082 | -.320** | -.241** | -.495** |  |  |
| Yoga Benefits | .187** | .190** | -.086 | -.076 | -.135** | .101* |  |
| Yoga Barriers | -.343** | -.330** | .333** | .288** | .379** | -.254** | -.357** |

Table B: Correlations between yoga dosage, psychosocial variables and perceived benefits and barriers to yoga for yoga students
