# Supplementary material for: ‘It is a lifeline’: International cross-sectional survey of benefits, barriers and acceptability of online yoga during the COVID-19 pandemic
Source: PLoS One. 2026 Feb 18;21(2):e0341852. doi: 10.1371/journal.pone.0341852 (PMC12915962; doi:10.1371/journal.pone.0341852)
Supplement: S2 Table — (DOCX) [file pone.0341852.s002.docx]

S2 Table. Results of linear regression models predicting psychosocial variables from demographic, yoga practice variables, and living situation and employment

| **Health and Step 1 Age well-being and gender**  **outcomes adjusted R^2^** | **Step 2 Yoga variables adjusted R^2^** | **Step 3 Living & Employment**  **adjusted R^2^** | **Final adjusted R^2^** | **Significant predictors** | **B** | **SE B** | **β** | **t** | **P value** |
| --- | --- | --- | --- | --- | --- | --- | --- | --- | --- |
| Depression .054 | .067 | .082 | .082 | Age  DPW  Living Situation  Employment | −0.129  −0.381  −2.202  -1.405 | 0.020  0.149  0.684  0.473 | −.223  −.104  −.097  -.090 | −6.539  −2.558  −3.220  -2.972 | <.001  .011  .001  .003 |
| Anxiety .085 | .089 | .090 | .090 | Age  Years practiced | -0.116  - 0.037 | 0.015  0.018 | -.265  -.069 | -7.796  - 2.008 | <.001  .045 |
| Stress .105 | .116 | .117 | .117 | Age | -0.157 | 0.020 | -.260 | -7.789 | <.001 |
| Soc Con .003 | .004 | .013 | .013 | Living Situation | 0.953 | 0.342 | .087 | 2.784 | .005 |
|  |  |  |  | Employment | 0.477 | 0.237 | .063 | 2.013 | .044 |
|  |  |  |  |  |  |  |  |  |  |

Variables in each step (Step 1: Age, Gender; Step 2: DPW, HPW, Years practiced; Step 3: Living situation, Employment); DPW = days/week; HPW = hours/week
